# Evaluating the prevalence of potentially inappropriate prescribing in older adults in intermediate care facilities: a cross-sectional observational study

**DOI:** 10.1007/s11096-017-0452-4

**Published:** 2017-03-17

**Authors:** Anna Millar, Carmel Hughes, Cristín Ryan

**Affiliations:** 1 School of Pharmacy, Queen’s University Belfast, 97 Lisburn Road, Belfast, BT9 7BL Northern Ireland, UK; 2 School of Pharmacy, Royal College of Surgeons in Ireland, 123 St. Stephens Green, Dublin 2, Ireland

**Keywords:** Aged, Inappropriate prescribing, Intermediate care facilities, Northern Ireland, Potentially inappropriate medication list

## Abstract

*Background* Potentially inappropriate prescribing (PIP) [encompassing potentially inappropriate medicines (PIMs) and potential prescribing omissions (PPOs)], is prevalent amongst older adults in primary and secondary care. However, PIP prevalence in intermediate care (IC) is unknown. *Objective* To determine the prevalence of PIMs/PPOs and associated patient factors. *Setting* Three IC facilities in Northern Ireland. *Method* The Screening Tool of Older People’s Prescriptions and the Screening Tool to Alert doctors to Right Treatment were used to identify PIP over 8 weeks. Wilcoxon signed-rank tests were performed to compare the prevalence of PIMs/PPOs at admission and discharge. Spearman’s correlation coefficients were calculated to determine factors associated with PIMs/PPOs (*p* < 0.05 considered significant). *Main outcome measure *Prevalence of PIMs/PPOs. *Results* 74 patients [mean age 83.5(±7.4) years] were included. Discharge medication data were available for 30 (40.5%) patients. 53 (71.6%) and 22 (73.3%) patients had ≥1 PIM at admission and discharge, respectively. 45 (60.8%) and 15 (50.0%) patients had ≥1 PPO at admission and discharge, respectively. No significant difference was found in PIM/PPO prevalence at admission compared to discharge (Z = −0.36, *p* = 0.72; Z = −1.63, *p* = 0.10). Increasing comorbidity and medication regimen complexity were associated with PIMs at admission (r = 0.265, *p* = 0.023; r = 0.338 *p* = 0.003). The number of medicines was correlated with PIMs at admission (r = 0.391, *p* = 0.001) and discharge (r = 0.515, *p* = 0.004). *Conclusion* Whilst IC represents an ideal setting in which to review prescribing, this study found PIP to be highly prevalent in older adults in IC, with no detectably significant change in prevalence between admission to and discharge from this setting.

## Impacts on practice

- Patients admitted to intermediate care facilities are generally older adults, prescribed polypharmacy, who had recently been discharged from hospital.
- Potentially inappropriate prescribing is highly prevalent amongst older adults in intermediate care and do not change significantly between admission and discharge.
- Intermediate care facilities provide an ideal setting in which to review the appropriateness of patients’ prescriptions, however this study suggests that does not occur.

## Introduction

In the United Kingdom (UK), intermediate care (IC) describes a range of services aimed at preventing unnecessary hospitalisation, promoting faster recovery from illness and maximising independence [1]. The development of IC has been driven largely in response to the increasing pressure faced by healthcare services as a result of the ageing population. Although the term ‘IC’ has its origins in the UK, several similar healthcare models exist elsewhere globally, including ‘sub-acute care’, ‘post-acute care’ and ‘transition care’ [2]. However, previous work has highlighted several inconsistencies between the concept of IC defined in the literature and the day-to-day realities of such services, for example the concept of IC preventing hospitalisation was not found to represent the reality in practice [3]. Additionally, despite the importance placed on the concept of multidisciplinary involvement in IC, the reality is that pharmacists are not widely involved with IC, nor is medicines management integral to IC services in Northern Ireland (NI) [3, 4]. Despite IC providing an ideal setting for medication review, concerns regarding the lack of responsibility for the review of patients’ medicines in IC has also been highlighted [3, 4].

Potentially inappropriate prescribing (PIP), encompassing potentially inappropriate medicines (PIMs) and the omission of clinically indicated medicines, i.e. potential prescribing omissions (PPOs), has received increasing attention due to its association with avoidable adverse drug events (ADEs) and hospitalisation amongst older adults [5]. Increasing age, comorbidity and number of medicines prescribed predispose individuals to PIP [6].

The Screening Tool of Older People’s Prescriptions (STOPP) and the Screening Tool to Alert doctors to Right Treatment (START) were developed to identify instances of PIMs and PPOs, respectively [7]. Published in 2008, STOPP/START (version 1) have been applied in numerous clinical settings to identify PIP in adults aged ≥65 years. The criteria have been recently updated [8]. STOPP/START version 2 now includes a total of 114 criteria, organised into 22 categories, representing a 31% increase in the number of criteria compared with version 1. Whilst predominantly an explicit set of criteria, STOPP version 2 also includes three implicit criteria within a new category (‘indication of medication’) which require clinical judgement in order to be applied to a patient’s medicines [8].

PIP has been shown to be prevalent amongst older adults in primary and secondary care settings, however, to the authors’ knowledge, no previous investigation into the prevalence of PIP in the IC setting has been conducted in the UK. Elsewhere in Europe, Bakken et al. [9] reported that the prevalence of inappropriate prescribing in IC increased from 24% at admission to 35% at discharge, as measured by the Norwegian General Practice (NORGEP) criteria, an explicit list of medicines, medicine dosages, and medicine combinations to be avoided in older adults [10].

## Aim of the study

As little was known about the IC population and the prevalence of PIP within it, the aim of the present study was to prospectively determine the PIP prevalence amongst older adults admitted to three IC facilities in NI over an 8-week period. The objectives of the study were to:

- Describe the IC population, including patient comorbidity, complexity of patients’ medication regimens and changes made to patients’ medications from admission to discharge
- Determine the prevalence of PIMs and PPOs using STOPP/START (version 2) at admission and discharge, and whether these changed significantly between these time-points
- Determine which (if any) patient-related factors (age, gender, comorbidity, medication regimen complexity, number of prescribed medications at admission and discharge) were associated with PIMs/PPOs.

## Ethics approval

Ethical approval was not required as the study was conducted as a ‘service evaluation’ within the Health and Social Care Trust governing the IC facilities. Approval for the study was granted by the Trust’s Audit Committee.

## Method

### Inclusion criteria

Patients were eligible for inclusion if they were:

- Aged ≥65 years at admission;
- Admitted to an IC-bed (i.e. not receiving other categories of care often provided within such facilities e.g. palliative care);
- Prescribed ≥1 regular (as opposed to ‘when required’) medicine.

Patients meeting the above criteria were included consecutively over the 8-week period (August–October 2014).

### Data collection

A data collection form was developed to record data from patients’ medical notes and prescription charts, including the following demographic information:

- Age
- Gender
- Pre-IC location (e.g. hospital, home)
- Reason for admission to IC

Where applicable, the date of discharge (to determine length of stay) and destination post-discharge were collected. Details of patients’ prescribed medicines, medical history and biochemical data were recorded to apply STOPP/START. As not all patients were discharged during the study, discharge data were not available for the entire cohort.

### Data analysis

Patient comorbidity was quantified using Charlson’s Comorbidity Index (CCI) [11], a widely used tool in clinical research [12]. Prescribed medicines were categorised according to the British National Formulary [13], a UK reference source, which categorises medicines according to their primary indication, e.g. ‘central nervous system (CNS)’ or ‘gastrointestinal system (GI)’. The complexity of patients’ medication regimens was quantified using the Medication Regimen Complexity Index (MRCI) [14]. The MRCI is a validated tool used to score a medication regimen based on the number of medicines, formulations, dosing frequencies and additional instructions (e.g. ‘take with food’) [14]. Changes made to patients’ medication regimens between admission and discharge were analysed descriptively as the number and type of changes made. Instances of PIP were identified by screening patients’ data collection forms against the full set of STOPP/START criteria. Data collection and screening was conducted by AM, a pharmacist experienced in the use of the STOPP/START criteria. The prevalence of PIMs and PPOs was defined as the percentage of patients with ≥1 PIM or ≥1 PPO, respectively.

### Statistical analysis

The Wilcoxon signed-rank test (Z) was performed to compare the prevalence of PIMs and PPOs at admission and discharge. Two-tailed bivariate correlations (Spearman’s rho correlation coefficient) were calculated to establish the relationship between the number of PIMs and PPOs at admission and discharge with: age, gender, CCI, MRCI, number of prescribed medications at admission and discharge. A probability value of <0.05 was considered significant. Statistical analysis was performed using Statistical Package for the Social Sciences (SPSS) version 20.0.

## Results

A total of 74 patients were included; 47 (63.5%) were female. The mean age was 83.5 years (±7.4; range 66–102). The majority of patients (68; 91.9%) were admitted to IC from hospital. Rehabilitation following a fall (with or without fracture) was the single most common reason for admission to IC, accounting for 26 (35.1%) admissions. Of the 74 admissions recorded, 38 (51.4%) patients were discharged during the 8-week period and three (4.1%) patients died (Table 1).

**Table 1:** Patient admission demographics

Demographics	Number of patient admissions n = 74 (%)
Gender	
Male	27 (36.5)
Female	47 (63.5)
Age at admission (years)	
Mean ± SD	83.5 (±7.4)
Range	66–102
CCI score	
0	14 (18.9)
1–3	38 (51.4)
4–6	20 (27.0)
7–9	2 (2.7)
Mean ± SD	2.49 (±2.08)
MRCI score	
Admission mean ± SD	26.5 ± 12.2
Discharge mean ± SD (n = 30)	26.1 ± 11.5
Source of admission	
Hospital	68 (91.9)
Home/usual place of residence	6 (8.1)
Length of stay (days) (n = 38)	
Mean ± SD	22.0 (±10.9)
Range	3–48
Discharge destination (n = 38)	
Own/family home	21 (55.3)
Nursing/residential care home	12 (31.6)
Hospital	5 (13.2)

*CCI* Charlson Comorbidity index; *MRCI* medication regimen complexity index

Patients were prescribed a mean of 10.4 (±3.8; range 3–19) regular medicines. Seventy-one (95.9%) patients were prescribed polypharmacy (≥4 medicines) [15] at admission. Data pertaining to medicines prescribed at discharge were available for 30 (40.5%) patients, who were prescribed a mean of 9.8 (±4.0; range 2–18) regular medicines.

A total of 30 patients’ medication data were collected from admission to discharge and overall 120 changes were made to these patients’ medication regimens during their stay in IC. A mean of 4.0 (±2.7; range 0–9) changes were made to patients’ medication regimens during their stay. Of these, 50 (41.7%) were medications being discontinued, 45 (37.5%) were medicines being started and 25 (20.8%) were ‘other’ changes (dose/frequency/formulation alterations).

### Prevalence of PIMs

STOPP identified 147 PIMs amongst 53 (71.6%) patients at admission (range 0–8). Thirty-four (72.3%) females had ≥1 PIM compared to 19 (70.4%) males. At discharge, 54 PIMs were identified amongst 22 (73.3%) patients (range 0–6). Seventeen (73.9%) females had ≥1 PIM compared to 5 (71.4%) males.

The 147 instances of PIMs at admission were attributable to 122 medications. The number of PIMs was greater than the number of associated medications as one medicine can be considered potentially inappropriate under ≥1 STOPP criteria. CNS medications accounted for the majority of medications responsible for PIMs at admission (50; 41.0%). The 54 instances of PIMs at discharge were attributable to 53 medications. GI system medicines accounted for the majority of medications responsible for PIMs at discharge (21; 39.6%).

The STOPP category ‘indication of medication’ was responsible for the majority of PIMs at both admission and discharge. Within this category, the STOPP criterion *‘any drug prescribed beyond the recommended duration’*, accounted for 14.3% of PIMs at admission and the STOPP criterion *‘any duplicate drug class prescription’*, accounted for 16.7% of PIMs at discharge (Table 2).

**Table 2:** The prevalence of PIMs at admission and discharge identified by STOPP (version 2)

STOPP criteria	Admission (n = 74)		Discharge (n = 30)	
	PIMs n (%)	Patients n (%)	PIMs n (%)	Patients n (%)
*Indication of medicine*				
Drug prescribed without evidence-based indication	6 (4.1)	6 (8.1)	5 (9.3)	5 (16.7)
Drug prescribed beyond recommended duration	21 (14.3)	19 (25.7)	7 (13.0)	6 (20.0)
Duplicate drug class prescription	18 (12.2)	17 (23.0)	9 (16.7)	7 (23.3)
*Cardiovascular system*				
Loop diuretic first-line for hypertension	3 (2.0)	3 (4.1)	–	–
Loop diuretic with urinary incontinence	3 (2.0)	3 (4.1)	–	–
Centrally-acting antihypertensives	1 (0.7)	1 (1.4)	–	–
ACE Inhibitors or ARBs with hyperkalaemia	1 (0.7)	1 (1.4)	–	–
*Antiplatelet/anticoagulant drugs*				
Aspirin, clopidogrel, dipyridamole, vitamin K antagonists, direct thrombin inhibitors or factor Xa inhibitors with significant bleeding risk	3 (2.0)	3 (4.1)	2 (3.7)	2 (6.7)
*CNS and psychotropic drugs*				
Tricyclic antidepressants with dementia, narrow angle glaucoma, cardiac conduction abnormalities, prostatism, or prior history of urinary retention	2 (1.4)	1 (1.4)	–	–
Benzodiazepines for ≥4 weeks duration	14 (9.5)	14 (18.9)	2 (3.7)	2 (6.7)
Antipsychotics (other than quetiapine or clozapine) in those with parkinsonism or Lewy body disease	–	–	1 (1.9)	1 (3.3)
Anticholinergics with delirium/dementia	5 (3.4)	5 (6.8)	2 (3.7)	2 (6.7)
Acetylcholinesterase inhibitors with persistent bradycardia, heart block or recurrent unexplained syncope or concurrent treatment with drugs that reduce heart rate	1 (0.7)	1 (1.4)	1 (1.9)	1 (3.3)
First-generation antihistamines	1 (0.7)	1 (1.4)	1 (1.9)	1 (3.3)
*GI system*				
PPI for uncomplicated peptic ulcer disease or erosive peptic oesophagitis at full therapeutic dosage for >8 weeks	6 (4.1)	6 (8.1)	3 (5.6)	3 (10.0)
Drugs likely to cause constipation in patients with chronic constipation where non-constipating alternatives are available	2 (1.4)	2 (2.7)	1 (1.9)	1 (3.3)
Oral elemental iron doses >200 mg daily	5 (3.4)	5 (6.8)	3 (5.6)	3 (10.0)
*Respiratory system*				
Theophylline as monotherapy for COPD	1 (0.7)	1 (1.4)	–	–
Systemic corticosteroids instead of inhaled corticosteroids for maintenance therapy in moderate-severe COPD	1 (0.7)	(1.4)	–	–
*Musculoskeletal system*				
Oral bisphosphonates with current or recent history of upper GI disease	1 (0.7)	1 (1.4)	–	–
*Urogenital system*				
Antimuscarinic drugs with dementia, or chronic cognitive impairment or narrow-angle glaucoma or chronic prostatism	6 (4.1)	6 (8.1)	3 (5.6)	3 (10.0)
*Endocrine system*				
Sulphonylureas with a long duration of action with type 2 diabetes mellitus	1 (0.7)	1 (1.4)	–	–
*Drugs that increase the risk of falls*				
Benzodiazepines	14 (9.5)	14 (18.9)	3 (5.6)	3 (10.0)
Neuroleptic drugs	5 (3.4)	5 (6.8)	2 (3.7)	2 (6.7)
Hypnotic Z-drugs	13 (8.8)	13 (17.6)	5 (9.3)	5 (16.7)
*Analgesic drugs*				
Use of oral or transdermal strong opioids as first line therapy for mild pain	1 (0.7)	1 (1.4)	–	–
Use of regular opioids without concomitant laxative	6 (4.1)	6 (8.1)	2 (3.7)	2 (6.7)
Long-acting opioids without short-acting opioids for break-through pain	2 (1.4)	2 (2.7)	1 (1.9)	1 (3.3)
*Anticholinergic drug burden*				
Concomitant use of ≥2 anticholinergic drugs	4 (2.7)	4 (5.4)	1 (1.9)	1 (3.3)

*ACE* Angiotensin converting enzyme; *ARB* angiotensin receptor blocker; *CNS* central nervous system; *GI* gastrointestinal; *PPI* proton pump inhibitor; *COPD* chronic obstructive pulmonary disease; *PIM* potentially inappropriate medicine

### Prevalence of PPOs

START identified 95 PPOs amongst 45 (60.8%) patients at admission (range 0–6 per patient). Twenty-nine (61.7%) females had ≥1 PPO compared to 16 (59.3%) males. At discharge, 34 PPOs were identified amongst 15 (50.0%) patients (range 0–6 per patient). Twelve (52.2%) females had ≥1 PPO compared to 3 (42.9%) males.

At both time-points, the most frequently identified PPOs related to the ‘musculoskeletal system’ START category, which accounted for 60.0% of PPOs at admission and 55.9% of PPOs at discharge (Table 3). The most frequent PPO at admission was *‘vitamin D supplement in older people who are housebound or experiencing falls or with osteopenia’* (20; 27.0%). The most frequent PPOs at discharge were *‘vitamin D and calcium supplements in patients with known osteoporosis’* (6; 17.6%), the omission of *‘bone anti*-*resorptive therapy in patients with documented osteoporosis’* (6; 17.6%), and *‘vitamin D supplement in older people who are housebound or experiencing falls or with osteopenia’* (6; 17.6%).

**Table 3:** The prevalence of PPOs at admission and discharge identified by START (version 2)

START criteria	Admission (n = 74)		Discharge (n = 30)	
	Patients n (%)	PPOs n (%)	Patients n (%)	PPOs n (%)
*Cardiovascular system*				
Vitamin K antagonists or direct thrombin inhibitors or factor Xa inhibitors with chronic atrial fibrillation	4 (4.2)	4 (5.4)	2 (5.9)	2 (5.9)
Antiplatelet therapy with history of coronary, cerebral or peripheral vascular disease	5 (5.3)	5 (6.8)	3 (8.8)	3 (8.8)
Antihypertensive therapy where systolic blood pressure consistently >160 mmHg and/or diastolic blood pressure consistently >90 mmHg; if systolic blood pressure >140 mmHg and/or diastolic blood pressure >90 mmHg, if diabetic	1 (1.1)	1 (1.4)	–	–
Statin therapy with a documented history of coronary, cerebral or peripheral vascular disease, unless the patient’s status is end-of-life or age is >85 years	6 (6.3)	6 (8.1)	3 (8.8)	3 (8.8)
ACE inhibitor with systolic heart failure and/or documented coronary artery disease	11 (11.6)	11 (14.9)	5 (14.7)	5 (14.7)
Beta-blocker with IHD	2 (2.1)	2 (2.7)	1 (2.9)	1 (2.9)
Appropriate beta-blocker with stable systolic heart failure	2 (2.1)	2 (2.7)	–	–
*Respiratory system*				
Regular inhaled β_2_ agonist or antimuscarinic bronchodilator for mild to moderate asthma or COPD	1 (1.1)	1 (1.4)	–	–
*Musculoskeletal system*				
DMARD with active, disabling rheumatoid disease	1 (1.1)	1 (1.4)	–	–
Bisphosphonates and vitamin D and calcium with long-term systemic corticosteroid therapy	3 (3.2)	3 (4.1)	1 (2.9)	1 (2.9)
Vitamin D and calcium supplement with osteoporosis and/or previous fragility fracture(s)	17 (17.9)	17 (23.0)	6 (17.6)	6 (17.6)
Bone anti-resorptive or anabolic therapy with documented osteoporosis, where no pharmacological or clinical status contraindication exists and/or previous history of fragility fracture(s)	16 (16.8)	16 (21.6)	6 (17.6)	6 (17.6)
Vitamin D in older people who are housebound or experiencing falls or with osteopenia	20 (21.1)	20 (27.0)	6 (17.6)	6 (17.6)
*Analgesics*				
Laxatives in patients receiving opioids regularly	6 (6.3)	6 (8.1)	1 (2.9)	1 (2.9)

*ACE* Angiotensin converting enzyme; *IHD* ischaemic heart diseases; *COPD* chronic obstructive pulmonary disease; *DMARD* disease modifying antirheumatic drug; *PPO* potential prescribing omission

### Change in prevalence of PIP between admission and discharge

No significant difference was found in the prevalence of PIMs at admission (median 1.5; interquartile range (IQR) 3.0) compared to discharge (median 2.0; IQR 3.0), Z = −0.36, *p* = 0.72. Similarly, no significant difference was found between PPO prevalence at admission (median 1.0; IQR 2.0) and discharge (median 0.5; IQR 2.0) Z = −1.63, *p* = 0.10.

### Patient-related factors associated with PIP

Increasing age was negatively correlated with PPOs at discharge (*r* = −0.436, *p* = 0.016). Increasing CCI was associated with PIMs at admission (*r* = 0.265, *p* = 0.023), as was increasing MRCI scores at admission (*r* = 0.338, *p* = 0.003). Increasing number of prescribed medicines at admission was associated with PIMs at both admission (*r* = 0.391, *p* = 0.001) and discharge (*r* = 0.515, *p* = 0.004).

## Discussion

To our knowledge, this was the first study investigating prescribing appropriateness amongst older adults in IC, in the UK. The average age, mortality, comorbidity and polypharmacy reported in this cohort, demonstrates that IC facilities cater to an older population in relatively poor health. This provides further evidence to support previously highlighted disparities between the concept of IC and the realities of the service [3]. For one, the conceptual role of IC in ‘preventing unnecessary hospitalisation’ is not currently being realised, as only a minority of patients were admitted to IC from their place of usual residence.

Using STOPP [8], this study found that the prevalence of PIMs amongst IC patients was 71.6% at admission and 73.3% at discharge. The majority of medicines responsible for PIMs at admission and discharge were CNS and GI medicines, respectively. The STOPP category ‘indication of medicine’ accounted for the majority of PIMs at both time-points. Notably, nearly one fifth of patients in this cohort at admission were prescribed benzodiazepines for a duration of more than 4 weeks. The use of benzodiazepines in older adults has been associated with clinically important ADEs including impaired cognition and falls [16]. Despite this, prescribing patterns of benzodiazepines are often in conflict with prescribing recommendations [17]. Patients may be reluctant to discontinue certain PIMs, including benzodiazepines, due to dependence associated with these medicines [18]. Prescribers may also be hesitant to discontinue medicines, particularly when initiated by another prescriber [18].

Using START [8], this study found that 60.8 and 50.0% of patients in the cohort had ≥1 clinically indicated medicines omitted from their medication regimen without a documented reason, at admission and discharge, respectively. The START category ‘musculoskeletal’ accounted for the majority of PPOs at both time-points. This category encompasses the omission of vitamin D, calcium and bone anti-resorptive agents, medicines of particular importance, given the frailty of the population under investigation. The reasons for their omission are unclear. However, such is the nature of STOPP/START that there is greater opportunity for the identification of PPOs amongst patients whose past medical histories are documented comprehensively and greater opportunity for the identification of PIMs amongst patients who past medical histories lack detail.

The prevalence of PIMs and PPOs reported are similar to those found in older adults in hospital [19] and higher than those reported in primary care [6] and nursing homes [20]. However, these studies used STOPP/START version 1, and so their findings cannot be compared directly with those reported here. It is, nonetheless, possible that the higher prevalence rates of PIMs/PPOs reported here, compared with previous studies using STOPP/START is due, in part, to the extension of criteria in the updated version [8], notably, the inclusion of the three new implicit criteria under the category ‘indication of medication’:

- Drug prescribed without evidence-based indication
- Drug prescribed beyond recommended duration
- Duplicate drug class prescription.

Despite patients’ medication regimens undergoing an average of four changes, it was found that the prevalence of PIP did not change significantly between admission and discharge. Ideally, PIP prevalence should decrease during a patient’s stay, as the rehabilitative IC environment provides a (theoretically) ideal opportunity for the optimisation of all aspects of patients’ medication regimens, including prescribing appropriateness and regimen complexity. Bakken et al. [9] also reported that the prevalence of PIP in an IC facility in Norway did not change significantly between admission and discharge. The authors suggested that this was due to prescribers’ reluctance to instigate changes for patients recently discharged from hospital. Whilst the findings of the present study would suggest that PIP is not addressed in IC, it was not within the scope of this study to determine reasons for this. Cullinan et al. [21] identified several barriers to behaviour change amongst prescribers in relation to appropriate prescribing. These included limited information-technology infrastructure, insufficient pharmacy input and a lack of geriatric pharmacotherapy training for prescribers [21]. Additionally, Anderson et al. [22] suggested that ‘inertia’ amongst prescribers plays a key role in the appropriateness of prescribing. This describes the failure to intervene, because the unknown consequences that may be associated with the cessation of a medication are less ‘desirable’ than its continuation, particularly if the medicine was initiated by another prescriber [22].

Unsurprisingly, the number of prescribed medicines in a patient’s regimen was positively correlated with PIMs. The association between PIMs and polypharmacy has been highlighted repeatedly in the literature [19, 23, 24]. Correspondingly, increasing patient comorbidity and medication regimen complexity were associated with the identification of PIMs at admission. Medication regimens can be rationalised in ways that do not compromise the overall intended therapeutic effect. Elliot et al. [25] demonstrated that a pharmacist-led intervention aimed specifically at rationalising patients’ medication regimens by reducing their complexity can be successfully implemented in IC. Finally, it must be noted that whilst efforts to reduce polypharmacy (and/or medication regimen complexity) may seem synonymous with improving prescribing appropriateness, the distinction between appropriate polypharmacy and *inappropriate* polypharmacy is not always clear [26].

### Limitations

The small sample size obtained from one geographical area of NI limits the generalisability of the findings. Furthermore, owing to the small sample size and the associated risk of a type II error, the significance of the findings reported should be interpreted with caution. Prescribers were not given the opportunity to explain their prescribing decisions for individual patients. Incomplete documentation of patients’ current diagnoses and biochemical information in the IC notes may have led to a lower rate of reporting of PIP in some cases, or a higher rate of reporting in others (i.e. where a medicine was clinically indicated but the patient’s notes did not document the indication). Due to the cross-sectional observational nature of the study it was not possible to make causal inference with regards to patient-related factors and the prevalence of PIP in IC. STOPP/START also have inherent limitations to their use. There may be a difference between recommendations derived from evidence and what is in the individual patient’s best interest [27]. STOPP/START are designed to be a user-friendly screening tools to aid the identification of *potentially* inappropriate prescribing; as such, they are limited in their ability to account for each patient’s holistic needs.

## Conclusion

This study of PIP amongst older adults in IC facilities in NI has identified PIMs and PPOs in substantial proportions of patients at both admission to and discharge from the IC setting. IC patients are typically older adults with marked levels of comorbidity and complex medication regimens. IC provides an ideal setting to address PIP and other medicines management issues with a view to lowering the risk of avoidable ADEs and associated negative outcomes in this population.
